# Developing New Geomaterials: The Case of the Natural Rubber Latex Polymers in Soil Stabilization

**DOI:** 10.3390/ma18081720

**Published:** 2025-04-09

**Authors:** Jair Arrieta Baldovino, Kevin Cardenas Diaz, Jorge Martínez Royero, Rohonal Serrano Sierra, Yamid E. Nuñez de la Rosa

**Affiliations:** 1 Civil Engineering Program, Universidad de Cartagena, Cartagena de Indias 130015, Colombia; kcardenasd@unicartagena.edu.co (K.C.D.); jmartinezr3@unicartagena.edu.co (J.M.R.); rserranos@unicartagena.edu.co (R.S.S.); 2 Faculty of Engineering and Basic Sciences, Fundación Universitaria Los Libertadores, Bogota 110231, Colombia

**Keywords:** soil cementation, new geomaterials, natural rubber latex, clay stabilization, natural polymers

## Abstract

This study explores using natural rubber latex (NRL) as a sustainable polymeric additive to improve the mechanical performance of cement-stabilized soil–crushed limestone waste (CLW) mixtures for pavement base applications. The experimental program involved varying cement contents (3%, 6%, and 9% by weight of soil) and NRL replacement levels (10%, 15%, 20%, and 25% of an 18% optimum water content, as determined by the standard Proctor test) under two target dry unit weights (16.6 and 17.6 kN/m^3^) and curing periods of 7 and 28 days. Unconfined compressive strength (UCS) tests and stiffness (Go) measurements were performed, while microstructural developments were examined using scanning electron microscopy (SEM) and energy-dispersive spectroscopy (EDS). The results indicate that an optimal NRL replacement exists for each cement content, enhancing interparticle bonding through the formation of polymeric films that reduce porosity and improve the ductility of the matrix. However, excessive NRL was found to retard cement hydration and ultimately decrease strength. On average, a 28-day curing period produced a 38% increase in UCS over 7-day values, independent of the NRL dosage. Comparisons with literature standards, including the ASTM D4609 threshold of 345 kPa for field strength, confirm that the optimized mixtures meet and exceed the minimum performance requirements. These findings underscore the potential of NRL as a viable alternative to conventional synthetic latexes in sustainable pavement base materials.

## 1. Introduction

For several decades, organizations such as the United Nations have warned about the indiscriminate use of natural resources. In 2015, the UN introduced the 15 Sustainable Development Goals (SDGs) to reduce CO_2_ emissions in the atmosphere, counteract greenhouse gases, and slow global warming in the coming years. Undoubtedly, one of the industries that produces the most CO_2_ emissions is the cement industry [1,2]. According to Riplay et al. [3], cement production facilities contribute over 8% of global carbon dioxide (CO_2_) emissions, with approximately 60% of these emissions stemming from process-related activities and the remaining 40% from energy consumption. One of the most significant challenges is reducing these emissions, which is difficult as the civil construction industry continues to rapidly expand in major cities of developing and developed countries. Consequently, significant contributions have been made within civil engineering—particularly in materials and geomaterials—to develop materials that generate low CO_2_ emissions [4,5,6]. In geotechnical engineering, for example, cement is widely used for stabilizing expansive and problematic clays, as is lime. However, due to recent price increases, cement is increasingly used to stabilize pavement bases and subbases and in compacted fills for foundation reinforcement or slope stabilization. Therefore, the search for alternative cementitious and soil-stabilizing materials is an area of growing importance in civil engineering [7]. Stabilization refers to the process of enhancing the engineering properties of natural soils by incorporating binders and additives that improve strength, durability, and resistance to environmental degradation. In geotechnical engineering, this process aims to modify soil characteristics, such as plasticity, compaction, and moisture sensitivity, thereby increasing its load-bearing capacity and reducing susceptibility to deformation and erosion [8,9,10].

**Figure 1:**
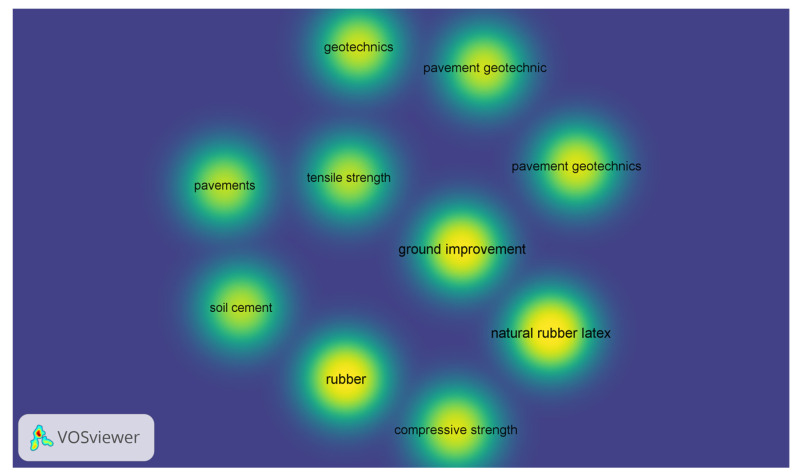
Density visualization.

**Figure 2:**
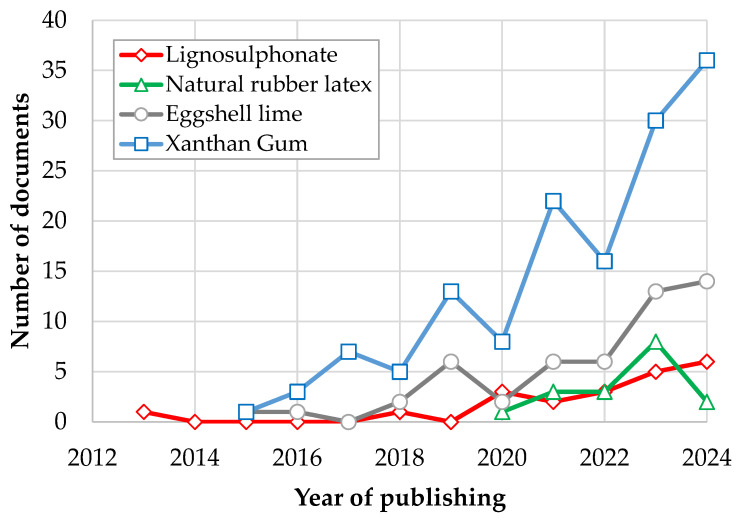
Number of documents by year.

To perform a systematic review analysis about use of NRL in soil stabilization, a search was conducted using SCOPUS (the abstract and citation database maintained by Elsevier). The SCOPUS search results in Table 1 reveal that while studies on natural rubber latex (NRL) as a soil stabilizer (15 results in 2023 and 17 in 2024) are fewer compared with those on xanthan gum and eggshell lime, the specialized research on NRL—as exemplified by the 15 detailed articles—demonstrates significant technical depth. Despite the lower publication volume indicated in the database, the focused and high-impact nature of NRL research underscores its potential as a sustainable and effective additive for soil stabilization. A density visualization of the keywords reported by studies in Table 1 is presented in Figure 1. The keywords more used in concordance with NRL–soil stabilization correlate with soil–cement, pavement, compressive strength, ground improvement, and tensile strength. The annual progression data in Figure 2 indicate distinct trajectories for each stabilizer compared with natural rubber latex (NRL). While NRL has a lower total publication count (17) relative to the other stabilizers, its rapid emergence post-2020 and subsequent fluctuations point to a specialized, high-impact niche that contrasts with the more consistent and extensive research seen for eggshell lime and xanthan gum.

Recent decades have seen a marked increase in the search for sustainable and cost-effective soil stabilization techniques, particularly for improving pavement bases and subbases. Among the various evaluated additives, natural rubber latex (NRL) has emerged as a promising “green” polymeric modifier due to its intrinsic elastic properties, excellent adhesion, and lower carbon footprint relative to conventional synthetic polymers [12,13]. Its widespread availability in many rubber-producing regions has enabled its successful incorporation into soil–cement and recycled aggregate systems, thereby enhancing mechanical performance, durability, and resistance to environmental degradation.

The literature indicates that integrating NRL into a soil system can significantly improve key mechanical properties. For instance, Buritatun et al. [12] investigated cement-stabilized soils modified with NRL for pavement base applications by varying cement contents (3%, 5%, and 7% by weight) and introducing NRL as a partial replacement for compacting water at ratios ranging from 10% to 30%. Their results revealed that an optimum NRL replacement exists for each cement content, with maximum improvements in unconfined compressive strength (UCS) achieved at 20%, 15%, and 10% NRL replacement for 3%, 5%, and 7% cement, respectively. Notably, for the lowest cement content, UCS enhancements reached up to 30%, and improvements in flexural strength (FS) were even more pronounced—up to 78% under optimal conditions. This suggests that the formation of continuous NRL films within the soil–cement matrix substantially improves interparticle bonding, although excessive NRL may retard cement hydration and, consequently, reduce overall strength. Complementary research by Yaowarat et al. [13] on concrete pavements modified with NRL supports these observations. By varying water-to-cement (*w*/*c*) ratios and dry rubber content-to-cement (*r*/*c*) ratios, their study found that while an increase in NRL content tends to reduce compressive strength, it markedly enhances flexural strength at specific *r*/*c* thresholds (0.58%, 1.16%, and 1.73% for *w*/*c* ratios of 0.3, 0.4, and 0.5, respectively). These findings underscore the dual role of NRL as both a strength-enhancing and ductility-improving agent, particularly under flexural loading conditions.

The enhancement of tensile properties is another critical aspect for pavement systems subjected to cyclic loading over extended service periods. Buritatum et al. [14] demonstrated that incorporating NRL into cement-stabilized soils increases indirect tensile strength (ITS) and significantly extends the fatigue life of the stabilized materials. Their experiments showed that NRL effectively reduces the accumulation of plastic strains under cyclic tensile loads, thereby delaying crack initiation and propagation through a crack-bridging mechanism enabled by a continuous and flexible NRL film within the cementitious matrix. In a subsequent study, Buritatum et al. [15] evaluated the fatigue performance of NRL-modified pavement bases under elevated curing temperatures. Testing specimens with cement contents of 3%, 5%, and 7% at temperatures of 25 °C, 40 °C, and 60 °C, they found that modified mixtures consistently outperformed conventional ones in terms of ITS, resilient modulus (Mr), and fatigue life (ITFL). The optimal NRL replacement ratios under these conditions mirrored those observed under ambient curing, indicating that NRL can mitigate the adverse effects of higher temperatures on early-age curing while reducing fatigue deterioration. Durability under environmental loading, especially in regions subject to repeated wetting–drying cycles, is also a major concern in soil stabilization. Buritatum et al. [16] investigated the durability of cement-stabilized pavement bases enhanced with NRL by subjecting samples to multiple wetting–drying cycles. Their findings indicated that NRL-modified specimens not only maintained higher UCS values but also experienced lower mass loss compared with control samples. This improvement is attributed to the cohesive film formed by NRL, which minimizes moisture ingress and inhibits the leaching of calcium ions from the cement matrix—a particularly beneficial attribute in tropical climates. Udomchai et al. [17] further confirmed that NRL-enhanced stabilization improves ITS, IT Mr, and ITFL under cyclic loading and wetting–drying conditions, effectively extending the service life of unpaved roads.

Emerging research has also explored the synergistic use of NRL with recycled materials and lateritic soils. Tran et al. [18] examined the partial replacement of lateritic soil with recycled aggregates—such as steel slag (SS) and recycled concrete aggregate (RCA)—while incorporating NRL as a modifier. By varying the dry rubber-to-cement (*r*/*c*) ratios, they demonstrated that the coexistence of cement hydration products and NRL films can significantly reduce pore spaces and enhance interparticle bonding, resulting in notable improvements in both UCS and ITS. However, they also cautioned that excessive NRL may inhibit cement hydration, leading to reduced mechanical development. Similarly, Nhieu et al. [19] focused on applying NRL in cement-stabilized RCA mixtures and found that it enhanced both UCS and ITS, contributing to a more homogeneous stress distribution within the matrix—an effect that helps mitigate early-age cracking and fatigue failure. Hoy et al. [20] further supported these findings by showing that NRL-modified blends of recycled materials and lateritic soils exhibit enhanced fatigue resistance and reduced brittleness, promoting a more sustainable and resilient pavement design.

Finally, NRL’s versatility extends beyond traditional soil stabilization. Its application in asphalt modification and geotextile reinforcement has also been documented. Aiamsri et al. [21] investigated asphalt concrete modified with pre-vulcanized NRL, identifying an optimal dry rubber content-to-asphalt cement ratio of 3% that improved Marshall stability, ITS, and resilient modulus, with additional benefits in fatigue life and rutting resistance. In a similar vein, Prongmanee et al. [22] demonstrated that coating water hyacinth-based geotextiles with NRL significantly enhanced their tensile strength and durability under repeated wetting–drying cycles. Moreover, Veena et al. [23] introduced an innovative approach for mitigating soil liquefaction by injecting NRL into sandy soils via a pressurized permeation method, which effectively reduced excess pore pressure generation and improved interparticle bonding and stiffness under cyclic loading.

Accordingly, this study investigates using NRL as a partial substitute for Portland cement in soil–cement–limestone powder mixtures, intending to reduce cement content while maintaining or enhancing key properties such as uniaxial compressive strength, flexural strength, and overall durability. By employing uniaxial compression tests, non-destructive ultrasonic pulse velocity assessments, and microstructural analyses via SEM, this research aims to determine the optimal NRL replacement ratio and elucidate the underlying microstructural mechanisms, thereby establishing NRL as a viable, sustainable alternative for geotechnical applications that contribute to reduced carbon emissions and improved environmental sustainability.

## 2. Materials and Methods

The experimental program initially comprised the collection, characterization, and preparation of the raw materials: clayey soil, crushed limestone waste (CLW), cement, and natural rubber latex (NRL). The soil was characterized through a series of tests, including chemical and mineralogical analyses, scanning electron microscopy (SEM), determination of Atterberg limits [24], grain-size distribution [25], specific gravity measurement [26], standard Proctor compaction [27], and color assessment. The particle size distribution (PSD) of soil tests were performed using a Bettersizer S3 Plus particle size analyzer (Dandong Bettersize Instruments Ltd, Dandong-China) (Particle Size Analysis Report), which has a measurement range of 0.01 μm to 3500 μm and was operated at 1600 rpm. The characterization of CLW involved evaluating its grain-size distribution under ASTM D2487, chemical composition, microscopy, and density. The chemical composition of the soil, cement, and CLW was determined through direct laboratory analysis using X-ray fluorescence (XRF) spectroscopy. The NRL was characterized based on the manufacturer’s specifications, and its fundamental properties—such as density and chemical composition—were also determined in the laboratory. After the characterization phase, the experimental plan was established. This plan involved defining the proportions of soil, CLW, NRL, and moisture in the mixtures to be compacted into cylinders measuring 10 cm in height and 5 cm in diameter and determining the curing times for the samples. Each mixture design was compacted in triplicate, producing three identical specimens for each mixture. Finally, after the curing period, the samples were subjected to uniaxial compression tests, ultrasonic pulse velocity measurements, and scanning electron microscopy (SEM).

### 2.1. Materials

**Figure 3:**
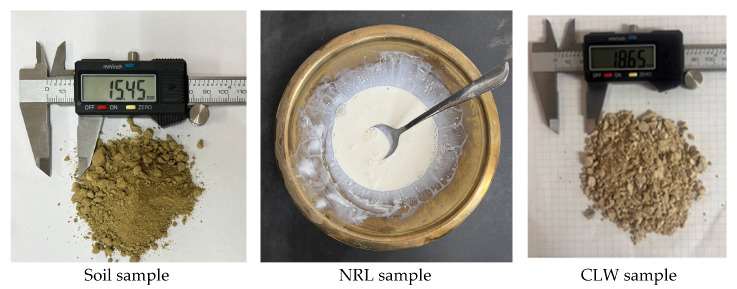
Raw material photos.

Figure 3 presents photographs of the raw materials, including the soil sample, natural rubber latex (NRL), crushed limestone waste, and an image of the compacted specimen. The soil sample used in this study was extracted from the Bayunca Formation in the northern area of Cartagena de Indias. The Bayunca Formation is characterized by sedimentary deposits formed in an intertidal environment, influenced by tidal channels rich in shells and clayey/sandy deposits filling abandoned channels with erosional contacts. The crushed limestone waste was extracted from a limestone quarry in San Juan Nepomuceno in the southern part of the Bolívar department, Colombia. Figure 3 shows the extracted CLW sample.

The natural rubber latex (NRL) used in this study was commercially acquired from Balonessupergolms Company in Bogotá, Colombia. NRL is a colloidal emulsion in which rubber particles are suspended in an aqueous serum containing a diverse array of dissolved organic and mineral compounds. Its unique properties—marked elasticity and exceptional resistance to water and electrical conduction—render it highly suitable for advanced applications, including soil stabilization. The employed NRL is a centrifuged, high-ammonium formulation with a minimum dry rubber content of 60% (*w*/*w*) and a total solids content ranging from 60.5% to 62.5%. It is characterized by a CAS number of 9003-31-0 and exhibits a boiling point of 100 °C in its vapor phase. Fully miscible in water, which facilitates this NRL’s incorporation into aqueous systems. The product is supplied as a white emulsion with a distinctive sweet, strongly ammoniacal odor. Additional specifications include a non-rubber content of no more than 2%, a pH of between 10.4 and 10.8, and a viscosity—measured using a Copa EZ #2 cup—ranging from 20 to 40 s. Figure 3 shows an NRL sample in a recipient.

**Figure 4:**
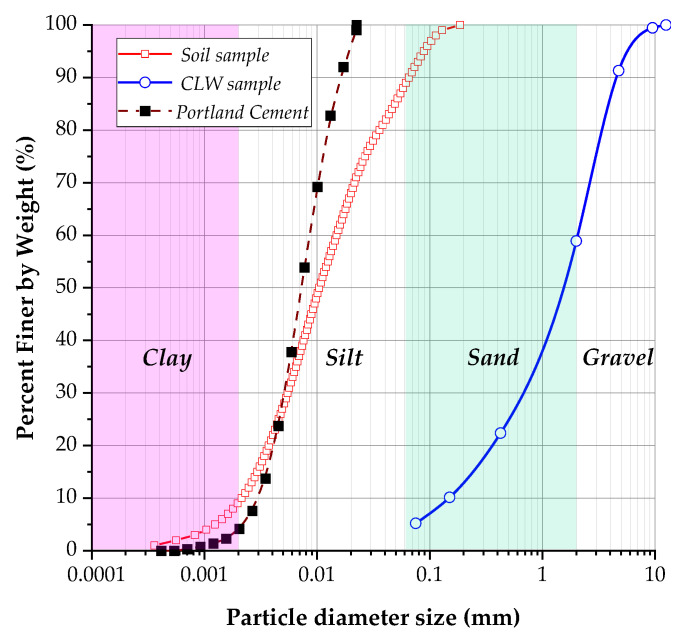
The granulometric curve of the soil sample, crushed limestone waste, and Portland cement.

The crushed limestone waste (CLW) used in this study was obtained from a limestone quarry located in San Juan Nepomuceno in the southern part of the Bolívar department, Colombia. The raw material was collected in its natural state and subsequently processed through standard crushing and sieving procedures to achieve the desired gradation for soil stabilization. Table 2 presents the characteristics and properties of the following raw materials: fine-grained soil and crushed limestone waste (CLW). The soil sample exhibited a liquid limit of 42% and a plasticity index of 15.9%, indicative of a plastic inorganic clay (USCS classification: CL) in concordance with ASTM D2487 [28]. The soil was predominantly composed of fine particles (Figure 4), with 82% silt and 10% clay, and had very small particle sizes, with a mean diameter (*d*_50_) of 0.011 mm and an effective diameter (*d*_10_) of 0.0021 mm. The uniformity coefficient of 7.14 suggested a moderate uniform gradation; its specific gravity was 2.80. Additionally, the soil sample was described as gray. In contrast, the CLW sample—crushed limestone waste—was non-plastic (NP); no liquid limit or plasticity index values were reported. Its grain-size distribution was significantly coarser, containing 10% gravel (4.75–76.2 mm), 30% coarse sand (2.00–4.75 mm), 38% medium sand (0.425–2.0 mm), 17% fine sand (0.075–0.425 mm), and 15% silt (0.002–0.075 mm), with no clay present (<0.002 mm). This graduation qualifies CLW as a well-graded sand (USCS classification: SW). The CLW sample had much larger particle sizes, with a *d*_50_ of 1.6 mm and a *d*_10_ of 0.15 mm, and its uniformity coefficient was notably higher at 13.67, accompanied by a coefficient of curvature of 1.59. Its specific gravity was 2.52, and a gray color characterized it.

The cement used in this study is a commercially available high early-strength cement. It exhibits compressive strengths of approximately 11–12 MPa at 1 day, 22–24 MPa at 3 days, and around 43.4 MPa at 28 days. These values demonstrate its rapid early strength development and ability to continue gaining strength over time, making it well-suited for applications requiring quick load-bearing capacity and long-term durability. Table 3 presents the chemical compositions by weight of the following raw materials—soil, cement, and CLW (crushed limestone waste). The soil sample was characterized by a high silica content (66.0% SiO_2_) and substantial alumina (21.1% Al_2_O_3_), with relatively low calcium oxide (3.0% CaO). In contrast, the cement exhibited a high CaO content (62.7%), along with moderate levels of SiO_2_ (21.1%), Al_2_O_3_ (5.2%), and Fe_2_O_3_ (2.6%), reflecting its calcium-rich binder composition. The CLW showed an even higher CaO concentration (72.4%) but with considerably lower amounts of SiO_2_ (9.0%) and AlO_3_ (1.3%), which is typical for a material derived from limestone. Notably, the CLW also contained elevated levels of MnO (14.3%) and P_2_O_5_ (2.1%), which may indicate the presence of phosphate minerals. The loss on ignition (LOI) values remained low across the samples—1.6% for the soil, 0.8% for the cement, and 2.1% for the CLW—suggesting minimal volatile content. These differences in chemical composition underscore the distinct mineralogical profiles of the materials, with soil being dominated by siliceous and aluminous constituents, cement acting as a calcium-based binder, and CLW providing a highly calcareous component with unique additional phases, which could influence its performance as a stabilizer in geotechnical applications.

**Figure 5:**
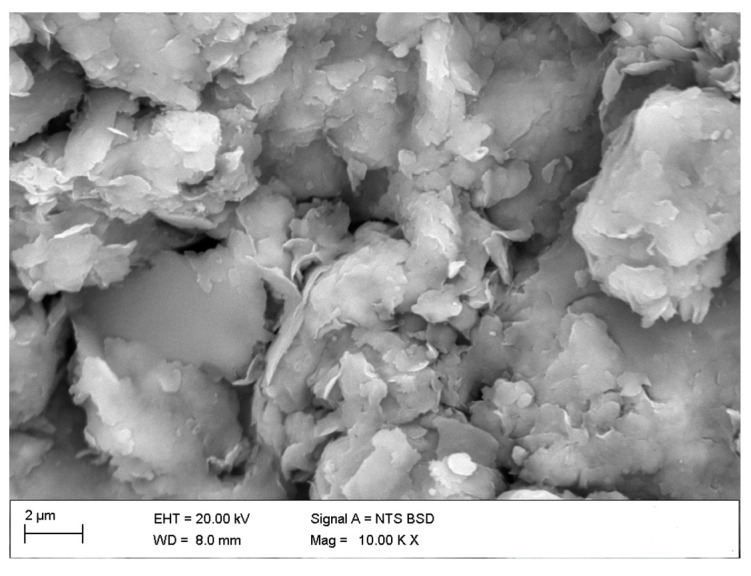
Microstructure of soil sample.

**Figure 6:**
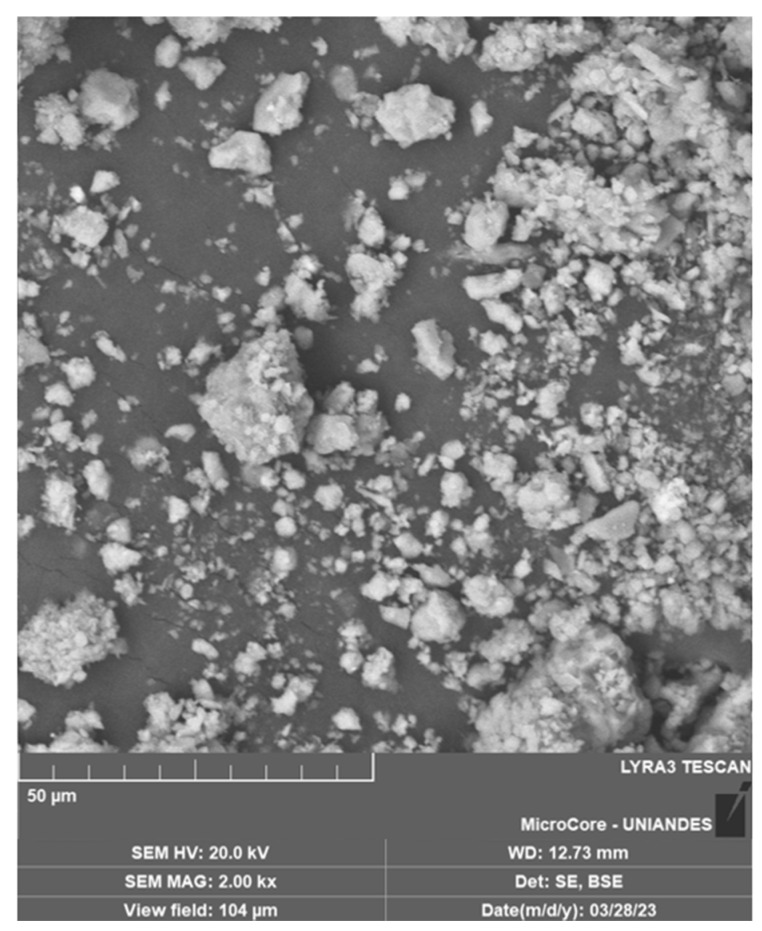
Microstructure of crushed limestone waste (CLW).

Figure 5 presents a microscopic image of a soil sample, while Figure 6 displays the microscopy of a crushed limestone waste (CLW) sample. The soil sample shows characteristic flocs typical of clay minerals, predominantly illite and kaolinite. In contrast, the CLW sample exhibits a variety of morphologies resulting from the crushing of limestone, primarily composed of calcium carbonate structures.

### 2.2. Methods

**Figure 7:**
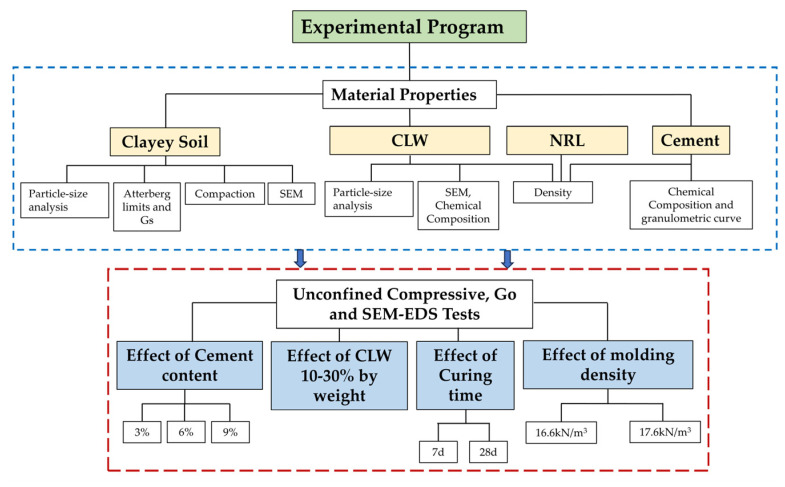
Flowchart of the experimental program.

After characterizing the raw materials, an experimental plan was developed to prepare mixtures of soil, cement, crushed limestone waste (CLW), natural rubber latex (NRL), and water. An optimum CLW content of 30% (by weight of soil) and a water content of 18%—as determined by Román Martínez et al. [29] via the standard Proctor test—were employed. NRL was incorporated by partially replacing the water at four levels (10%, 15%, 20%, and 25% of the water content); the NRL was first blended with water and then added to a dry mixture of soil, cement (at 3%, 6%, or 9% by weight of soil), and CLW. Specimens were prepared using a sub-compaction method, wherein each mixture was statically compacted in three layers within a cylindrical mold (10 cm in height and 5 cm in diameter) at target dry unit weights of 16.6 and 17.6 kN/m^3^, with each mixture being compacted in triplicate. The compaction procedure was that followed by American ASTM D698-12 [27]. Finally, the specimens were cured for 7 and 28 days. Table 4 summarizes the complete experimental plan for the compacted specimens. The experimental program of the study is summarized in Figure 7.

### 2.3. UCS and Stiffness (Non-Destructive) Program

**Figure 8:**
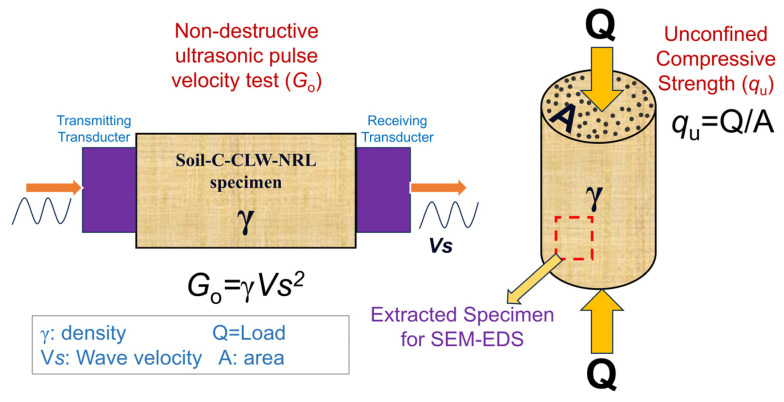
Test set-up of stiffness and unconfined compressive strength.

After the curing period, the small-strain stiffness test was performed on the specimens in accordance with ASTM C597-02 [30]. Ultrasonic transducers were positioned at each end of the specimen, and wave propagation velocity was measured using a Pundit PL-200 ultrasonic tester, (Proceq SA, Zurich, Switzerland), (see setup in Figure 8). This measurement was used to evaluate potential cracks or voids since the stiffness of a compact material is indicated by the maximum wave velocity traversing it—higher velocities reflect a more compact material that deforms less. The deformation of the specimen can be expressed as the ratio of its wet unit weight to the square of the wave velocity. The Pundit PL-200 provides a measurement resolution of 0.1 µs, operates at a pulse voltage between 100 and 450 V, and uses transducers with a nominal frequency of 54 kHz.

The unconfined compressive strength test (UCS) was conducted on each specimen after 7 and 28 days of curing (after ultrasonic non-destructive tests), in accordance with ASTM D2166-03 [31]. Each specimen was placed in a multi-test machine with a load capacity of up to 50 kN and controlled via dedicated software. An axial load was then applied at a constant displacement rate of 1.15 mm/s until failure occurred, and the ultimate load was recorded as the critical parameter for evaluating the mechanical strength of the specimens.

### 2.4. Microstructural Analysis

After the uniaxial compressive strength (UCS) and non-destructive ultrasonic pulse tests, intact specimens with a volume of 1 cm^3^ were carefully extracted from selected samples for microstructural analysis via SEM using the Tescan Lyra 3, TESCAN ORSAY HOLDING, Brno—Kohoutovice, Czech Republic. The specimens were prepared by precisely cutting and obtaining representative cubes (Figure 8), then dried at 60 °C for 24 h to eliminate residual moisture without altering the microstructure. Following drying, the samples were mounted on aluminum stubs using conductive carbon tape and, if necessary, sputter-coated with a thin layer of gold to enhance surface conductivity and imaging quality. SEM imaging was conducted at an acceleration voltage of 15–20 kV, utilizing both secondary and backscattered electron detectors to capture high-resolution morphological details. Concurrently, real-time EDS analysis was performed to determine the elemental composition across various regions of interest, with instrument settings optimized to achieve a resolution of approximately 0.1 µm. This integrated SEM–EDS methodology provided comprehensive insights into the microstructural integrity and elemental distribution within the stabilized soil–cement–latex–CLW mixtures, elucidating interfacial interactions between the binder and aggregate phases.

### 2.5. Statistical Analysis

The mechanical test results were statistically analyzed using SPSS Statistics Version 26 at a significant level of 5% (*p* < 0.05). An analysis of variance (ANOVA) was performed to assess the significance of the primary factors—cement content, NRL replacement level, target dry unit weight, and curing time—and their interactions, as well as to verify any potential curvature in the response behavior. This univariate linear analysis was used to determine whether the mean values of unconfined compressive strength (UCS) and stiffness (Go) significantly differed among the various experimental groups.

## 3. Results and Discussions

### 3.1. Effects of NRL on the Unconfined Compressive Strength of Soil–Cement–CLW Compacted Blends

**Figure 9:**
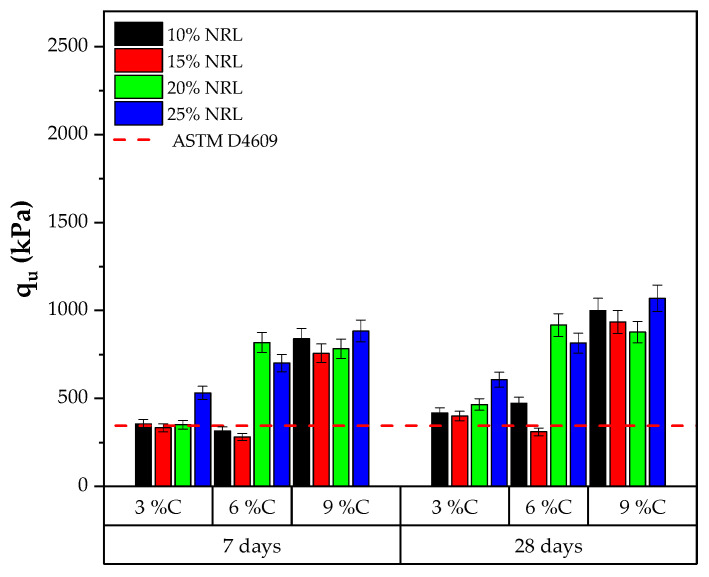
Results of unconfined compressive strength considering a molding dry unit weight of 16.6 kN/m^3^.

**Figure 10:**
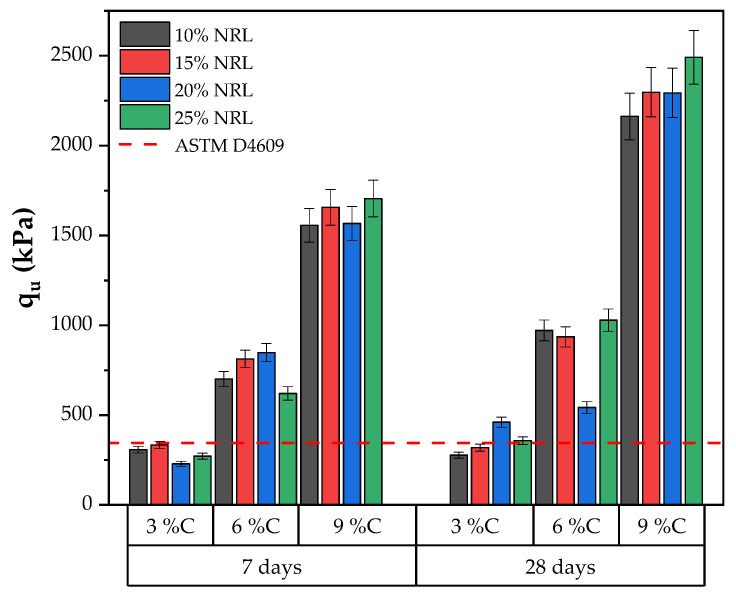
Results of unconfined compressive strength considering a molding dry unit weight of 17.6 kN/m^3^.

The uniaxial compressive strength (UCS) results of the soil–cement–CLW–NRL mixtures were evaluated under varying conditions of target dry unit weight, cement content, and NRL replacement levels with curing periods of 7 and 28 days, as presented in Figure 9 and Figure 10. For mixtures compacted at a dry unit weight of 16.6 kN/m^3^ with 3% cement, the UCS increased from approximately 355 kPa at 10% NRL replacement to 532 kPa at 25% replacement after 7 days and from 418 kPa to 607 kPa after 28 days, indicating that, at a low cement content, higher NRL replacement improves strength. In contrast, mixtures with 6% cement at 16.6 kN/m^3^ showed a marked peak in UCS at a 20% NRL replacement level (approximately 817 kPa at 7 days and 917 kPa at 28 days), while a further increase to 25% slightly reduced the strength. Mixtures with 9% cement under the same density conditions exhibited significantly higher strengths, with 28-day UCS values ranging from around 877 kPa at 20% NRL to 1070 kPa at 25% NRL replacement. When the target dry unit weight was increased to 17.6 kN/m^3^, the effect of cement content became more pronounced; for instance, the 9% cement mixtures achieved 28-day UCS values exceeding 2160 kPa, reaching up to 2491 kPa at 25% NRL replacement (Figure 10). These results suggest that the optimal NRL replacement level depends on the cement content and target density: while NRL enhances interparticle adhesion and improves mechanical performance by forming polymeric films that fill pore spaces, excessive NRL can retard cement hydration and, in some cases, lower strength. This delicate balance between improved bonding and hydration retardation is consistent with previous findings [12,13,15], underscoring the need to optimize NRL content to maximize the performance of cement-stabilized soil mixtures.

**Figure 11:**
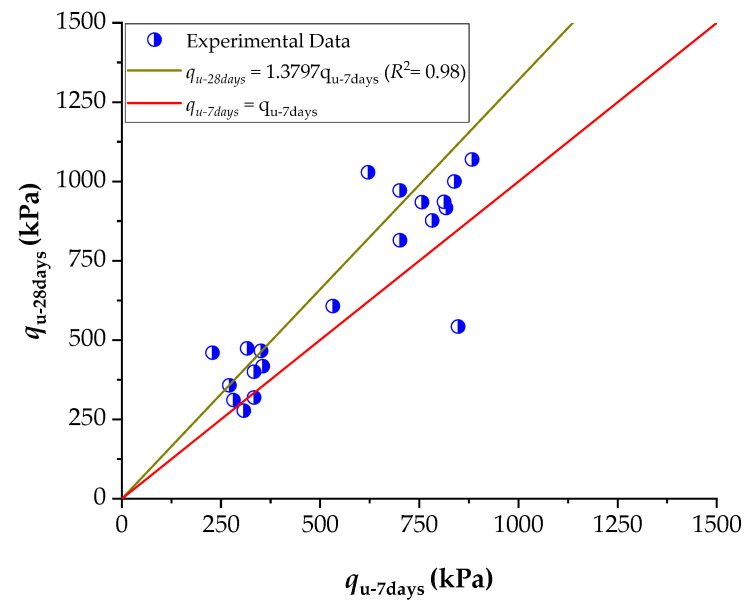
Increment of unconfined compressive strength at 28 days of curing in relation to 7 days of curing.

When the compaction density was increased to 17.6 kN/m^3^, a similar pattern was observed, albeit with some variations. For instance, at 9% cement and 7 days, UCS values exceeded 1550 kPa at 10% NRL replacement and continued to rise with increasing NRL until around 25% replacement, reaching approximately 1705 kPa. However, in some cases—particularly at lower cement contents—the UCS decreased with excessive NRL replacement, likely due to the retardation of cement hydration, a phenomenon also reported by Buritatun et al. [12]. Figure 11 shows the average increase in UCS between 7 and 28 days of curing across all mixtures. On average, the UCS at 28 days was approximately 38% higher than that measured at 7 days, regardless of the NRL replacement level.

Comparing these findings with the literature, Buritatun et al. [12] demonstrated that the optimum NRL replacement ratios can lead to UCS improvements of up to 30% for a low cement content (3%), with the percentage of improvement diminishing as the cement content increases. The present results corroborate this behavior, with relative enhancements in UCS being more notable in mixtures with 3% cement than those with 9% cement. Similarly, Nhieu et al. [19] reported that NRL can enhance the UCS of cement-stabilized recycled concrete aggregate mixtures, with the optimum dry rubber-to-cement ratio critical for maximizing strength. The data indicate that an optimal NRL dosage exists, beyond which further increases result in a decline in UCS, emphasizing the need to balance improved interparticle bonding provided by NRL films with the potential retardation of cement hydration.

According to ASTM D4609 [32], an unconfined compressive strength (UCS) of 345 kPa is the minimum threshold for adequate field strength in soil stabilization, as presented in Figure 9 and Figure 10. The UCS results from the present study demonstrate that, under a target dry unit weight of 16.6 kN/m^3^, many mixtures surpass this threshold even at 7 days of curing. For instance, the 3% cement mixtures yielded UCS values of approximately 355 kPa at 10% NRL replacement and 532 kPa at 25% replacement, while the 6% and 9% cement mixtures reached even higher UCS values, with the 9% cement mixtures achieving values near 840 kPa to 883 kPa at 7 days. Similar trends were observed at 28 days, where UCS values further increased, confirming enhanced strength development over time. These results indicate that incorporating NRL, when optimized concerning cement content and replacement level, effectively improves the soil–CLW matrix to meet and exceed the ASTM D4609 criterion for adequate stabilization. This finding is consistent with previous studies [15], which also reported that an optimum NRL dosage enhances UCS by improving interparticle bonding and reducing porosity, thus ensuring that the stabilized mixtures possess sufficient strength for field applications.

### 3.2. Effects of NRL on the Stiffness of Soil–Cement–CLW Compacted Blends

**Figure 12:**
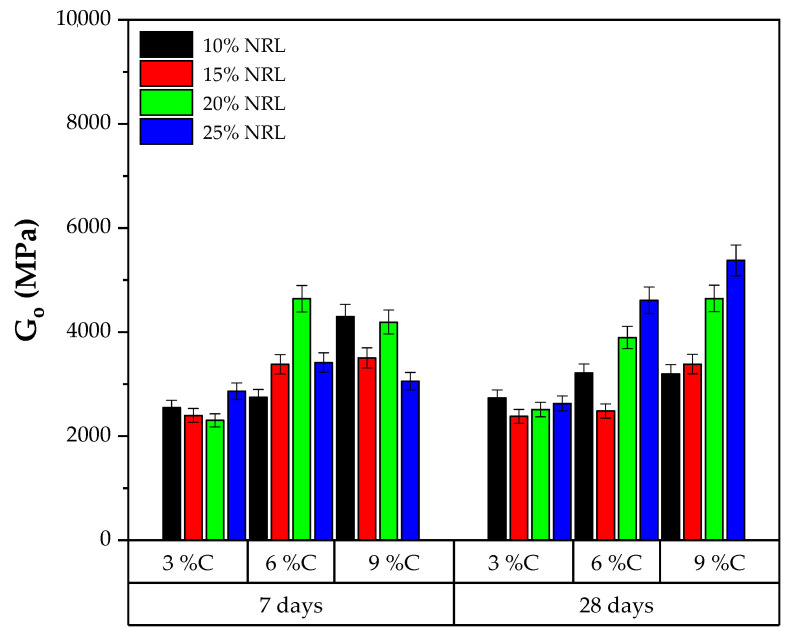
Results of stiffness considering molding dry unit weight of 16.6 kN/m^3^.

**Figure 13:**
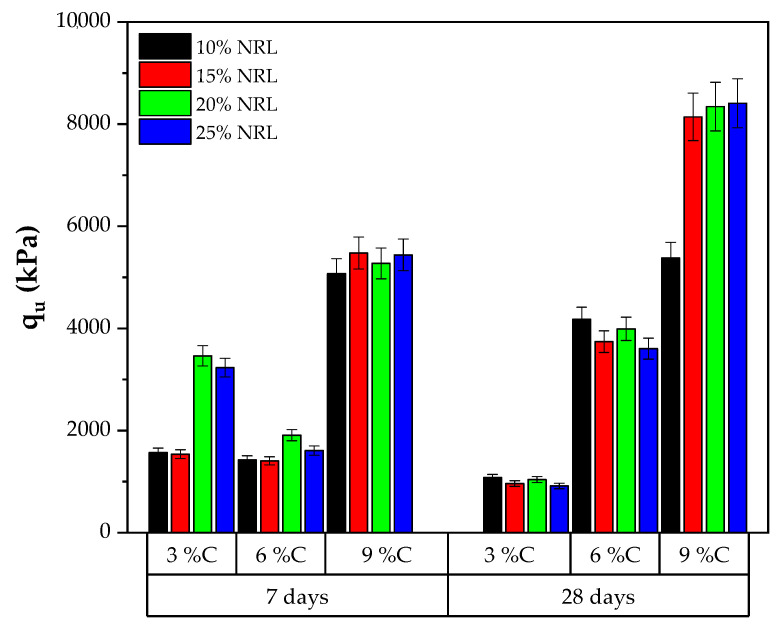
Results of stiffness considering molding dry unit weight of 17.6 kN/m^3^.

The stiffness parameter (Go) in the soil–CLW–NRL mixtures substantially varied with cement content, NRL replacement level, curing time, and target dry unit weight, as presented in Figure 12 and Figure 13. At the lower compaction density (16.6 kN/m^3^), the 7-day Go values ranged roughly from 2300 to 4600 MPa, generally increasing with both cement content and NRL dosage—although excessive NRL (e.g., 25% replacement) can, in some cases, retard cement hydration and reduce stiffness. For instance, at 3% cement and 7 days, Go rose from about 2303 MPa at 20% NRL to 2862 MPa at 25% NRL, whereas at 6% cement, it peaked at approximately 4641 MPa (20% NRL), highlighting the interplay between NRL films and cement hydration products. By 28 days, most mixtures at 16.6 kN/m^3^ showed further increases in Go, reflecting ongoing hydration and improved microstructural development. Notably, the 9% cement mixtures often exhibited the highest Go values, underscoring the dominant role of binder content in enhancing stiffness.

When the compaction density was increased to 17.6 kN/m^3^ (Figure 13), a more pronounced gain in Go was observed, particularly at higher cement contents and longer curing times. For example, at 9% cement and 28 days, Go exceeded 8000 MPa for 15–25% NRL replacement, with the highest recorded value being 8410 MPa at 25% NRL. This significant jump reflected the combined effects of improved particle packing (due to higher density), greater cement hydration, and the latex-induced polymer network that bridges microvoids and cracks. However, at lower cement contents or suboptimal NRL levels, the Go values remained comparatively lower, illustrating that a delicate balance between latex dosage, cement hydration, and compaction is necessary to maximize stiffness. Overall, the data confirm that higher cement contents, increased curing times, and well-chosen NRL replacement levels—especially under adequate compaction—lead to a denser, more cohesive matrix, thereby elevating the stiffness of stabilized materials.

### 3.3. Relationship Between Stiffness and Unconfined Compressive Strength

**Figure 14:**
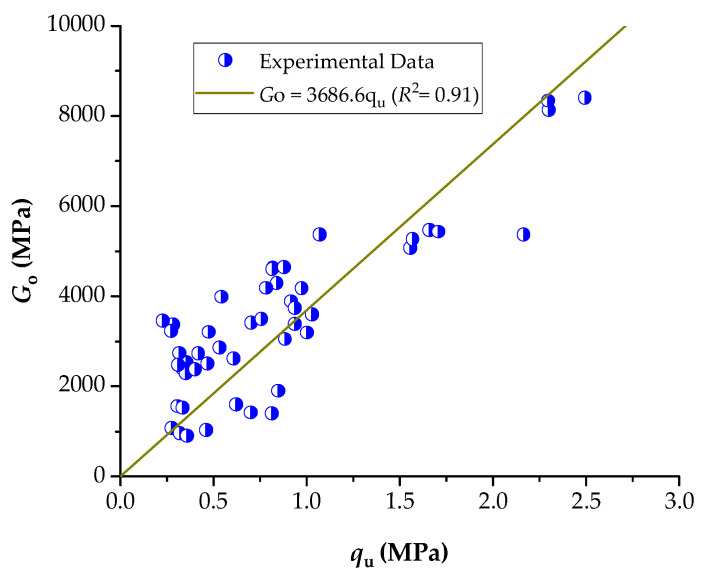
The direct relationship of unconfined compressive strength and stiffness.

The analysis of the compacted soil–cement–NRL–CLW mixtures revealed a strong linear correlation between the uniaxial compressive strength (q_u_) and the stiffness parameter (Go), which the following equation can express: Go = 3686.6q_u_ (*R*^2^ = 0.91) (Figure 14). This equation indicates that approximately 91% of the variability in Go is explained by changes in q_u_. The slope of 3164.3 implies that even small increases in qu lead to significant enhancements in the material’s stiffness. In these mixtures, the combined effect of cement hydration products and the natural rubber latex (NRL) network produces a denser, more cohesive matrix with improved interparticle bonding. Consequently, qu, as determined by uniaxial compression tests, can be a reliable predictor of a mixture’s stiffness (Go). This strong correlation is valuable for optimizing mix designs in soil stabilization applications, as it enables adjustments in component proportions to achieve the desired mechanical performance and long-term durability.

The analysis of the UCS (q_u_) and stiffness (Go) results reveals several key insights into the behavior of the soil–cement–CLW–NRL mixtures. At a target dry unit weight of 16.6 kN/m^3^, for example, mixtures with 3% cement exhibited a moderate UCS increase with NRL replacement up to 25%, where qu rose from approximately 355 kPa at 10% NRL to 532 kPa at 25% NRL. This increase was accompanied by a corresponding change in stiffness, with Go values indicating a favorable enhancement of the composite matrix. In contrast, at higher cement contents (6% and 9%), the optimal NRL replacement appeared to differ; for instance, the 6% cement mixtures reached a peak UCS of roughly 817 kPa at 20% NRL replacement, while the 9% cement mixtures exhibited higher absolute strength values yet a less pronounced dependence on NRL dosage. Moreover, the results obtained at a higher compaction density of 17.6 kN/m^3^ generally display higher UCS values, particularly in the 9% cement mixtures, where qu exceeded 1550 kPa even at lower NRL percentages. This trend suggests that increased compaction enhances the formation of cement hydration products, thereby partially mitigating the hydration-retarding effects of excessive NRL. The interrelationship between qu and Go further demonstrates that improvements in UCS are strongly linked to the corresponding stiffness enhancement, reinforcing the concept that even small increases in compressive strength can result in significant gains in stiffness. These observations align with previous findings by Buritatun et al. [12] and Nhieu et al. [19], who also reported that an optimal balance exists between NRL replacement and cement content. In these studies, the beneficial effects of NRL—namely improved interparticle bonding and reduced porosity—were maximized at specific replacement levels, beyond which further increases in NRL began to impede cement hydration and reduce overall strength. Thus, the present results underscore the critical importance of optimizing NRL dosage, cement content, and compaction density to achieve the desired mechanical performance and durability in sustainable pavement base materials.

Table 5 summarizes the direct relationship between the stiffness (Go) and strength (UCS) of various compacted geomaterial blends as reported in the literature. In the present study, the Go/UCS index was 3686.6 with an *R*^2^ of 0.91, which indicates a strong linear correlation between stiffness and compressive strength in the soil–cement–CLW–NRL mixtures. When compared with similar blends, the clayey soil–cement–CLW blend reported by Román Martínez et al. [29] exhibited a higher Go/UCS index of 4828.8 with an *R*^2^ of 0.96, suggesting that, for that particular system, the stiffness increased more sharply with strength. Similarly, the sand–cement system in [33] showed an even higher index of 7465.9 (*R*^2^ = 0.86), reflecting a more pronounced stiffness sensitivity to strength in sand-based mixes. Interestingly, materials incorporating polymers, such as clayey soil–xanthan gum in [11], yield a much lower index of 1915.3 (*R*^2^ = 0.98), which may be attributed to the distinct rheological and bonding characteristics imparted by xanthan gum compared with cementitious binders. Other blended systems, such as clayey soil–glass-powder–cement [10] with an index of 2909.68 (*R*^2^ = 0.97) and various sand–ground-glass–carbide lime systems [34,35] with indices ranging from 985.34 to 30,690, demonstrate that the stiffness-to-strength relationship is highly dependent on the specific materials and curing conditions involved.

### 3.4. Microstructure of Compacted Blends

Using scanning electron microscopy (SEM) combined with energy-dispersive spectroscopy (EDS), a detailed analysis of the microstructure of the compacted soil–RPC–cement–natural rubber latex mixtures was conducted. This methodology allowed for high-magnification imaging that provided an in-depth understanding of the internal structure and composition of the specimens. The analysis revealed critical information regarding the interactions among the soil, crushed limestone waste (RPC), cement, and natural rubber latex, including forming a cohesive matrix, developing binder films, and distributing key elemental constituents. Specific morphological features—such as well-bonded interfaces between particles, the homogeneous dispersion of the RPC, and the integrity of the latex coatings—were identified, offering a solid basis for explaining the mechanical behavior and durability of the mixtures under varying environmental and loading conditions.

**Figure 15:**
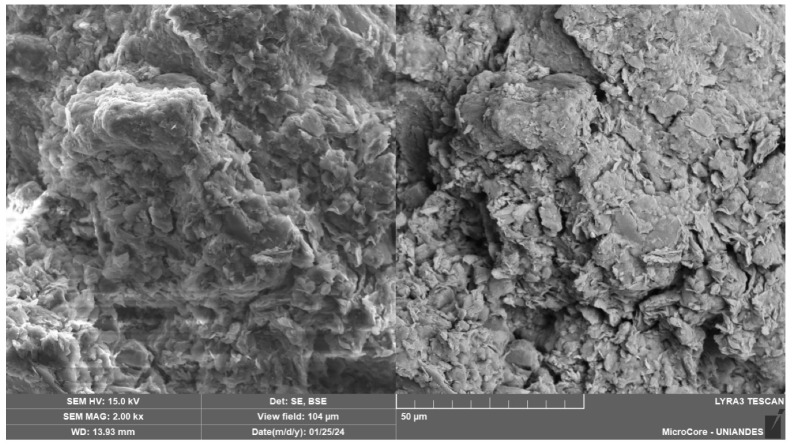
Microstructure image of soil–cement–CLW–NRL compacted sample (magnification 2000×) using 3% cement, 30% CLW, and 15% NRL replacement.

**Figure 16:**
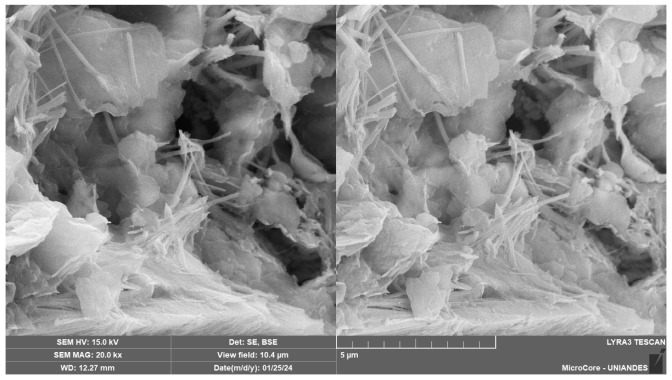
Microstructure image of soil–cement–CLW–NRL compacted sample (magnification 20,000×) using 6% cement, 30% CLW, and 20% NRL replacement.

**Figure 17:**
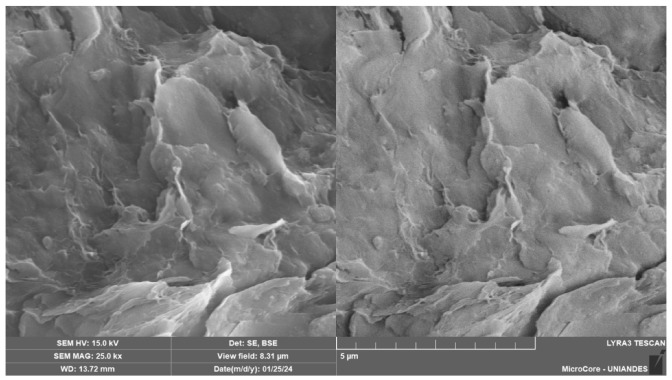
Microstructure image of soil–cement–CLW–NRL compacted sample (magnification 25,000×) using 6% cement, 30% CLW, and 25% NRL replacement.

**Figure 18:**
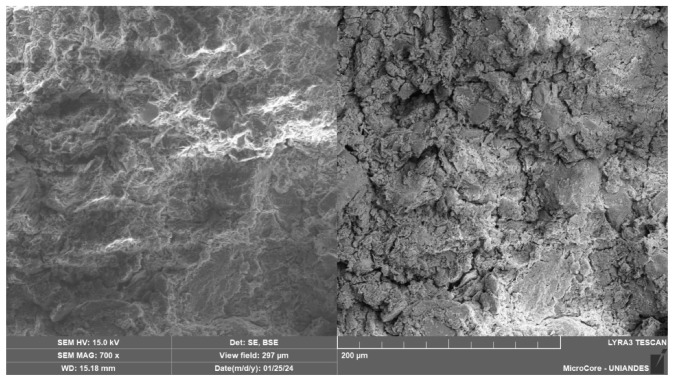
Microstructure image of soil–cement–CLW–NRL compacted sample (magnification 700×) using 9% cement, 30% CLW, and 20% NRL replacement.

**Figure 19:**
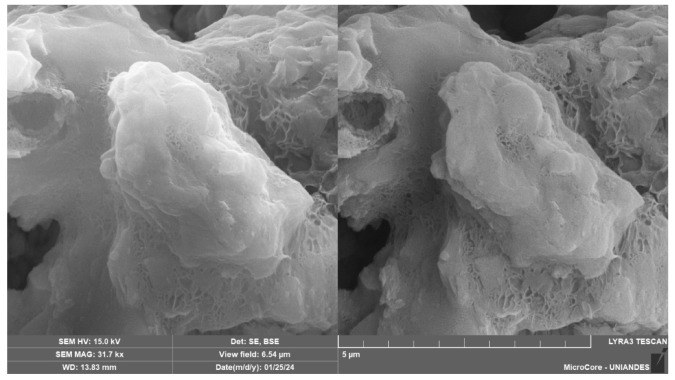
Microstructure image of soil–cement–CLW–NRL compacted sample (magnification 31,700×) using 6% cement, 30% CLW, and 10% NRL replacement.

The SEM analysis of the soil–cement–CLW–NRL mixtures reveals a complex and highly integrated microstructure that varies with cement content and NRL replacement levels, as evidenced by Figure 15, Figure 16, Figure 17, Figure 18 and Figure 19. Figure 15, at 2000× magnification, shows a dense and well-bonded matrix in a sample with 3% cement, 30% CLW, and 15% NRL replacement, where the cement hydration products—likely C–S–H and ettringite—are uniformly distributed throughout the pore spaces, forming a continuous network that binds the soil and CLW particles. This observation aligns with Buritatun et al. [12], who noted that a dense cementitious matrix significantly improves mechanical strength. In Figure 16, at 20,000× magnification, the sample with 6% cement, 30% CLW, and 20% NRL replacement exhibits a notable presence of needle-like ettringite crystals embedded within a more refined microstructure; these crystals enhance early-age strength by filling voids, a phenomenon reported by Horpibulsuk et al. [36]. Figure 17, at 25,000× magnification, presents an even finer view of a sample with 6% cement, 30% CLW, and 25% NRL replacement, where the natural rubber latex manifests as a pronounced, continuous polymeric film that infiltrates the pore spaces; this film appears thicker and more interconnected, indicating an excess of NRL that may begin to hinder cement hydration, a finding consistent with the adverse effects of high NRL dosages described by Yaowarat et al. [13].

Conversely, Figure 18, captured at 700× magnification for a sample with 9% cement, 30% CLW, and 20% NRL replacement, shows a relatively coarser but still well-integrated matrix; the higher cement content promoted the formation of abundant hydration products, yet the presence of NRL films is still evident, albeit in a less dominating fashion, which suggests that at higher cement levels, the cementitious bonding plays a more significant role, as also observed by Tran et al. [18]. Finally, Figure 19, at 31,700× magnification, reveals the microstructure of a sample with 6% cement, 30% CLW, and 10% NRL replacement, where a well-developed calcium silicate hydrate (C–S–H) network is visible; the lower NRL content here allowed for optimal cement hydration, resulting in a robust and finely interlocked matrix, which supports the findings by Hoy et al. [37] that an optimal NRL dosage can enhance mechanical properties without impeding hydration. These images (SEM Figure 15, Figure 16, Figure 17, Figure 18 and Figure 19) illustrate the crucial balance between cement content and NRL replacement. While appropriate NRL incorporation improves interparticle adhesion through polymer film formation and reduces porosity, excessive NRL can retard hydration, whereas too little NRL fails to enhance bonding sufficiently. This detailed microstructural characterization thus confirms that an optimal NRL replacement level exists for maximizing the mechanical performance and durability of cement-stabilized soil mixtures.

### 3.5. Statistical Analysis of Influence of Cement and NRL in the Strength and Stiffness of Compacted Blends

The ANOVA for q_u_ (Table 6) indicates that the overall model was highly significant with an *R*^2^ of 0.621. The cement content exhibited a strong effect, while the target dry unit weight (γd) approached significance and NRL showed no significant impact (*p* = 0.776). Similarly, the ANOVA for Go (Table 7) shows a significant overall model (*p* = 0.000 and *R*^2^ = 0.572), with cement content again being the dominant factor (*p* < 0.000), while γd (and NRL did not significantly contribute. Notwithstanding these statistical outcomes, the experimental data reveal that certain levels of NRL replacement are associated with improved mechanical performance. For instance, at a target density of 16.6 kN/m^3^ with 3% cement, the UCS increased from approximately 355 kPa at 10% NRL to 532 kPa at 25% NRL replacement after 7 days. Similarly, at higher cement contents (6% and 9%), optimal NRL levels corresponded to notable peaks in UCS, although the effect became less pronounced as cement content increased. These trends suggest that, while cement content is the primary determinant of both compressive strength and stiffness, the incorporation of NRL may enhance interparticle bonding and ductility—effects that are reflected in the observed strength gains over time (with an average UCS increase of 38% from 7 to 28 days) and in the strong correlation between UCS and stiffness.

## 4. Conclusions

This study demonstrates that incorporating natural rubber latex (NRL) into cement-stabilized soil–crushed limestone waste (CLW) mixtures markedly enhances their mechanical performance. For mixtures compacted at 16.6 kN/m^3^ with 3% cement, the 7-day unconfined compressive strength (UCS) increased from approximately 355 kPa at 10% NRL replacement to 532 kPa at 25% replacement, while at 28 days, the UCS improved from 418 kPa to 607 kPa. Similarly, at a cement content of 6%, the optimum NRL replacement (around 20%) yielded a 7-day UCS of about 817 kPa and a 28-day UCS of approximately 917 kPa, whereas mixtures with 9% cement reached UCS values of roughly 840–883 kPa at 7 days. At a higher target density of 17.6 kN/m^3^, 9% cement mixtures exhibited even greater strengths, with UCS values exceeding 1550 kPa at 7 days and reaching up to 1705 kPa at optimal NRL levels. Across all mixtures, extending the curing period from 7 to 28 days resulted in an average UCS increase of 38%, underscoring the importance of continued cement hydration in developing long-term strength.

These findings indicate that NRL improves the soil–cement–CLW matrix by forming polymeric films that enhance interparticle bonding, reduce porosity, and improve ductility. However, excessive NRL replacement tends to retard cement hydration, leading to a decline in strength. Significantly, even the lowest UCS values measured in this study far exceed the ASTM D4609 minimum threshold of 345 kPa, confirming that the stabilized mixtures are effective for field applications. Overall, the results emphasize the critical need to balance cement content, NRL dosage, and compaction density to optimize sustainable pavement base materials’ mechanical performance and durability.

This study was conducted under controlled laboratory conditions, which may not fully capture the variability and complexity of field environments, including the effects of fluctuating temperatures, moisture conditions, and long-term cyclic loading. Additionally, the range of mix designs and curing times explored was relatively limited, potentially restricting the generalizability of the results to a wider variety of soils and operational conditions. Future research should address these aspects by incorporating extended testing regimes and field trials to further validate the applicability of the proposed stabilization approach.

## Figures and Tables

**Table 1:** Search results for several documents depend on a selected search string in the SCOPUS database (from 2013 to 2024). Continued by Baldovino et al. [11].

Stabilizer	Scopus String	Total Results 2023	Total Results 2024
Lignosulphonate	(“soil stabilization” OR “soil improvement” OR “ground improvement”) AND (“Lignosulphonate”)	15	21
Natural rubber latex	(“soil stabilization” OR “soil improvement” OR “ground improvement”) AND (“natural rubber latex” OR “natural latex”)	15	17
Eggshell lime	(“soil stabilization” OR “soil improvement” OR “ground improvement”) AND (“eggshell” OR “egg lime”)	37	51
Xanthan Gum	(“soil stabilization” OR “soil improvement” OR “ground improvement”) AND (“xanthan”)	105	141

**Table 2:** Characteristics and properties of the soil sample and CLW. NP, non-plastic. CL, inorganic clay. SW, well-graded sand.

Property	Unit	Soil	CLW
ASTM D4318 [24] Limit Liquid, L.L.	%	42.0	*NP*
ASTM D4318 [24] Plasticity Index, P.I.	%	15.9	*NP*
ASTM D854 [26] Specific Gravity, Gs	-	2.80	2.52
ASTM D2487 [28] Gravel (4.75–76.2 mm)	%	0	10
ASTM D2487 [28] Coarse Sand (2.00–4.75 mm)	%	0	30
ASTM D2487 [28] Medium Sand (0.425–2.0 mm)	%	0	38
ASTM D2487 [28] Fine Sand (0.075–0.425 mm)	%	8	17
ASTM D2487 [28] Silt (0.002–0.075 mm)	%	82	15
ASTM D2487 [28] Clay (<0.002 mm)	%	10	0
ASTM D2487 [28] Mean Diameter (*d*_50_)	mm	0.011	1.6
ASTM D2487 [28] Effective Diameter (*d*_10_)	mm	0.0021	0.15
ASTM D2487 [28] Uniformity Coefficient *C*_u_	-	7.14	13.67
ASTM D2487 [28] Coefficient of Curvature *C*_c_	-	0.96	1.59
Skempton Activity of Clay	-	1.60	-
USCS Classification	-	CL	SW
Color	-	Gray	Gray

**Table 3:** Chemical composition of the soil sample, cement, and CLW.

Compound	Concentration by Weight in %		
	Soil	Cement	CLW
CaO	3.0	62.7	72.4
MgO	-	3.8	2.1
SiO_2_	66.0	21.1	9.0
Al_2_O_3_	21.1	5.2	1.3
Fe_2_O_3_	0.9	2.6	0.9
TiO_2_	0.3	-	-
K_2_O	3.1	-	-
SO_3_	4.0	3.5	-
Na_2_O	-	0.1	-
MnO	-	0.2	14.3
P_2_O_5_	-	-	2.1
LOI	1.6	0.8	2.1

**Table 4:** Mixed proportion design for compacted blends of soil, cement, CLW, NRL, and water.

Molding *γ*_d_ (kN/m^3^)	Soil	Cement	CLW	NRL Replacement in Water (%)	Curing Times (Days)	Specimens
16.6	100	3	30	10, 15, 20 and 25	7, 28	24
	100	6	30	10, 15, 20 and 25	7, 28	24
	100	9	30	10, 15, 20 and 25	7, 28	24
17.6	100	3	30	10, 15, 20 and 25	7, 28	24
	100	6	30	10, 15, 20 and 25	7, 28	24
	100	9	30	10, 15, 20 and 25	7, 28	24

**Table 5:** The direct relationship between stiffness and strength of different geomaterials reported in the literature.

Type of Compacted Blend	Go/UCS Index	*R* ^2^	Reference
Present study	3686.6	0.91	-
Clayey soil–cement–CLW	4828.8	0.96	[29]
Sand–cement	7465.9	0.86	[34]
Clayey soil–xanthan gum	1915.3	0.98	[11]
Clayey soil–glass-powder–cement	2909.68	0.97	[10]
Sand–ground-glass–carbide lime (7 days)	21,690	0.99	[34]
Sand–ground-glass–carbide lime (180 days)	30,690	0.98	[34]
Osorio sand–glass-powder–carbide lime	2169.49	0.94	[35]
Rio Pardo sand–glass-powder–carbide lime	1785.74	0.85	[35]
Porto Alegre sand–glass-powder–carbide lime	985.34	0.82	[35]

**Table 6:** ANOVA table for the q_u_ results.

Source	Sum of Squares	Degrees of Freedom	Mean Squares	*Z*	*p*-Value	Significance (*p*-Value < 0.05)
Corrected Model	10,002,384.224 *	6	1,667,064.037	11.200	<0.000	yes
Cement (C)	9,344,460.009	2	4,672,230.004	31.391	<0.000	yes
γd	493,252.683	1	493,252.683	3.314	0.076	no
NRL	164,671.532	3	54,890.511	0.369	0.776	no
Error	6,102,492.353	41	148,841.277			
Total	49,411,208.833	48				

* Corrected *R*^2^ = 0.621.

**Table 7:** ANOVA table for the stiffness Go results.

Source	Sum of Squares	Degrees of Freedom	Mean Squares	*Z*	*p*-Value	Significance (*p*-Value < 0.05)
Corrected Model	87,108,980.020 ^a^	6	14,518,163.337	9.120	0.000	yes
Cement (C)	78,074,538.949	2	39,037,269.474	24.522	0.000	yes
γd	4,142,980.079	1	4,142,980.079	2.603	0.114	no
NRL	4,891,460.993	3	1,630,486.998	1.024	0.392	no
Error	65,268,720.310	41	1,591,920.008			
Total	737,724,685.315	48				

^a^ Corrected *R*^2^ = 0.572.

## Data Availability

The original contributions presented in this study are included in the article. Further inquiries can be directed to the corresponding authors.
